# Retroperitoneal invasive fibromatosis after laparoscopic radical resection of colon cancer: a case report and literature review

**DOI:** 10.3389/fonc.2025.1582253

**Published:** 2025-07-17

**Authors:** Tiantian Shan, Yiqi Zhang, Yang Yao, Min Li, Xiaoying Li

**Affiliations:** ^1^ Department of Cardiology, The Affiliated Hospital of Qingdao University, Qingdao, China; ^2^ Department of Emergency, Central Hospital Affiliated Shandong First Medical University, Jinan, China; ^3^ Department of Emergency, Jinan Central Hospital, Jinan, China; ^4^ Medical College, Yangzhou University, Yangzhou, China

**Keywords:** invasive fibromatosis, retroperitoneum, sigmoid colon cancer, imaging study, case report

## Abstract

Invasive fibromatosis is a rare, locally invasive, benign fibrous tumor that mainly occurs in the anterior abdominal wall and limbs, with a lower incidence in the retroperitoneum. This article reports a case of retroperitoneal invasive fibromatosis in a 63-year-old male patient. The patient underwent resection for sigmoid colon cancer and was diagnosed with retroperitoneal invasive fibromatosis through enhanced CT, pathological examination and Whole Exome Sequencing one year after surgery. Given the rarity of postoperative surgery-related retroperitoneal invasive fibromatosis in patients with colorectal cancer, this article aims to provide a reference for clinical research on retroperitoneal invasive fibromatosis by reporting this case.

## Introduction

Invasive fibromatosis, also known as desmoid-type fibromatosis (DF), is a rare, locally aggressive, benign fibrous tumor that originates from a mesenchymal cell line and is a subtype of mesenchymal tumor (1, 2). This tumor is a rare soft tissue sarcoma (STS), representing only 3% of all STSs and 0.03% of all tumors, with a high likelihood of recurrence without metastasis (3, 4). The local recurrence rate is high, ranging from 18% to 56% (5). DF usually occurs sporadically, but approximately 5% of cases are related to familial adenomatous polyposis (FAP); it most often occurs in the anterior abdominal wall and limbs and rarely occurs in the retroperitoneum (6, 7). The exact etiology is unclear. These tumors are rarely symptomatic, but when symptomatic, it is mainly due to the impact of the mass on the adjacent vasculature and organs. In most cases, they are found incidentally during imaging studies (7).

We report a case of a retroperitoneal invasive fibroma that was incidentally diagnosed. The patient is a 63-year-old man with a history of resection for sigmoid colon cancer who presented to the emergency department complaining of recurrent abdominal pain. In this case, we discuss the characteristics of this extremely rare tumor.

## Case presentation

**Figure 1:**
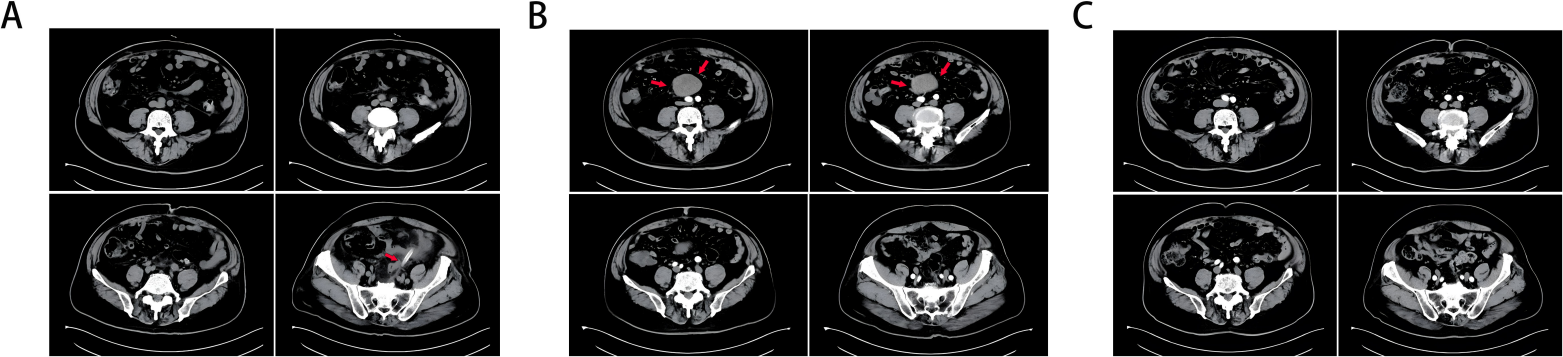
**(A)** CT taken 1 day after colon cancer surgery, and an arrow marks the drainage tube. **(B)** A retroperitoneal tumor (marked with an arrow) was found on intensive CT 1 year after colon cancer surgery, and there was vascular nourishment in the tumor. **(C)** Abdominal CT 3 years after resection of colon cancer and 2 years after resection of invasive fibroids, and no obvious abnormalities were found.

A 63-year-old man was found to have neoplastic polyps measuring approximately 2.2*1.4*1 cm in the sigmoid colon during routine gastrointestinal endoscopy. The polyps were removed via ESD. Pathology revealed moderately differentiated tubular adenocarcinoma with an SM invasion depth ≥ 1000 µm. The possibility of submucosal invasion was not excluded. Laparoscopic resection of colon cancer was performed at the Department of Gastroenterology, and approximately 24 cm of the sigmoid colon was removed. Pathology revealed that there was no atypical hyperplasia. **Figure 1A** shows the postoperative CT image.

Within six months postsurgery (February–August 2021), the patient developed persistent symptoms, including increased stool frequency, loose stools, and weight loss. Gastrointestinal endoscopy performed in August 2021 revealed normal gastrointestinal mucosa without tumor recurrence, polyps, or anastomotic stricture. By December 2021, the patient’s bowel habits and stool morphology had normalized. In February 2022, the patient experienced lower abdominal discomfort and mild abdominal pain. Contrast-enhanced CT (**Figure 1B**) revealed a retroperitoneal mass with a vascular supply, suggesting possible tumor recurrence.

**Figure 2:**
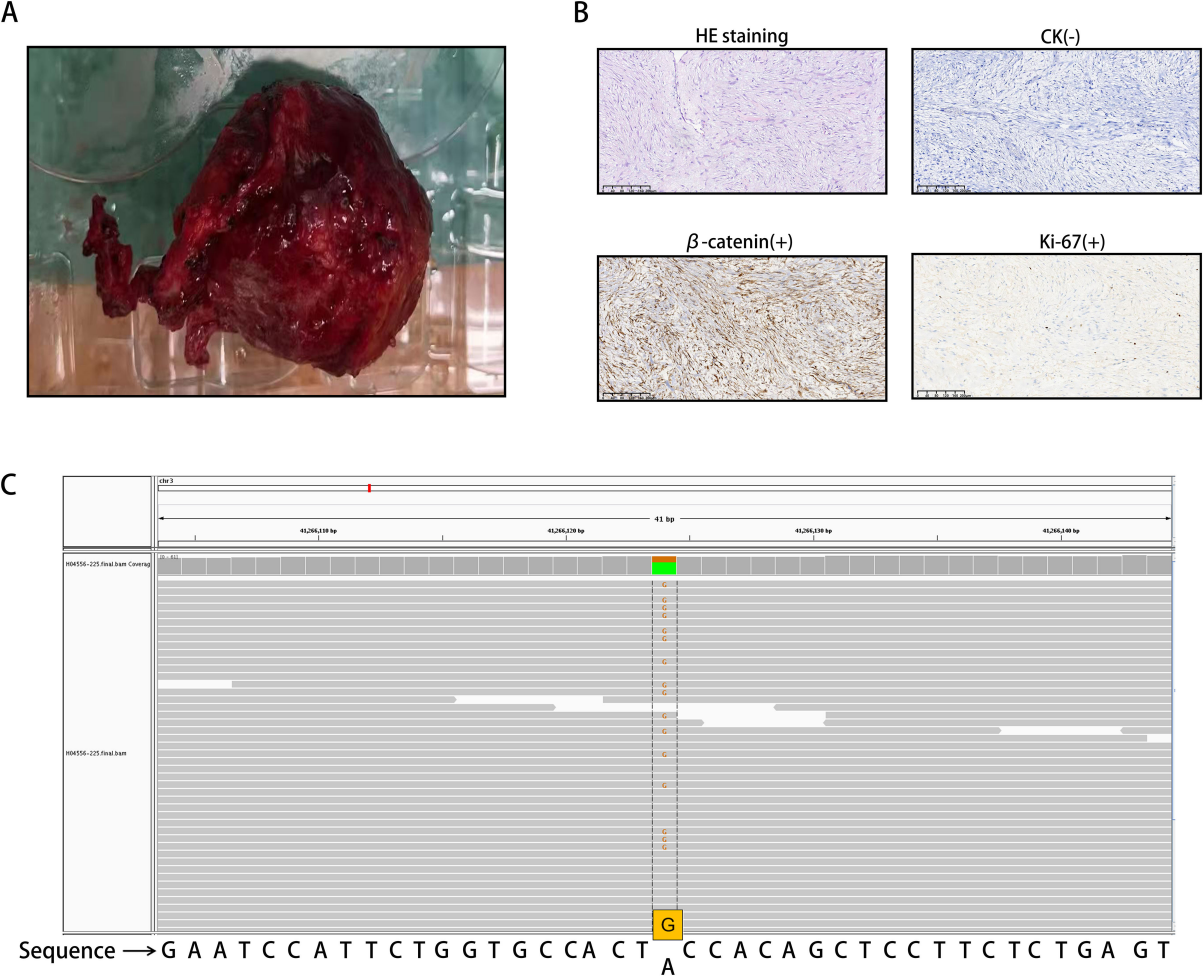
**(A)** Invasive fibroid surgery and excised tumor tissue. **(B)** Spindle cell neoplasia with ill-defined demarcation from surrounding adipose tissue on HE staining. The cells were sparse and dense and exhibited interstitial collagen degeneration, mucus degeneration, and high erythrocyte extravasation. The immunohistochemical staining results were as follows: Ki-67 (2%+), β-catenin (partial nucleus +), and CK (-). **(C)** The CTNNB1 gene mutation is located on chromosome 3, with mutation coordinates chr3:41266124 (hg19). The 121st nucleotide corresponding to exon 3 of the coding region changes from A to G (c.A121G). This was determined through the use of WES.

Since the possibility of tumor recurrence could not be ruled out, the patient underwent laparoscopic exploration and retroperitoneal tumor resection. The tumor (**Figure 2A**) was excised intraoperatively and sent for histopathological analysis. Under the microscope, spindle cell tumor-like hyperplasia was observed on HE stained sections, and the boundary with the surrounding adipose tissue was unclear. The results of immunohistochemical staining revealed the following: β-catenin (+), vimentin (+), Ki-67 (2%), SMA (-), ALK (-), MDM2 (-), CDK4 (-), CD34 (-), Bcl-2 (-), STAT-6 (-), S-100 (-), SOX-10 (-), CD117 (-), DoG-1 (-), and CK (-). The HE staining and immunohistochemical results suggested that the mass was invasive fibromatosis and a borderline tumor (**Figure 2B**).

To confirm the diagnosis of tumor slices, whole exome sequencing (WES) was performed (**Figure 2C**). We found a CTNNB1 gene mutation on chromosome 3, with mutation coordinates chr3:41266124 (hg19). The 121st nucleotide corresponding to exon 3 of the coding region changes from A to G (c.A121G). This mutation is a missense mutation in all transcripts, causing the 41st amino acid of CTNNB1 encoded protein β-catenin to mutate from threonine to alanine (p.T41A). Based on the above results, the patient was diagnosed with DF.

The patient recovered uneventfully, with resolution of symptoms. During the 2-year follow-up period (March 2022–December 2024), the patient remained asymptomatic with normal bowel function, appetite, and weight restoration to preoperative levels. Serial abdominal CT scans (**Figure 1C**) confirmed that there was no recurrence of the retroperitoneal fibromatosis or colorectal carcinoma.

## Discussion

Fibromatosis is a disease characterized by fibrous connective tissue hyperplasia and it can be divided into primary fibromatosis and invasive fibromatosis (8). Invasive fibromatosis, also known as desmoid-type fibromatosis (DF), is a type of interstitial neoplasm formed by adherent cells, fascia and aponeurosis. In terms of location, invasive fibromatosis can be divided into several groups: extraabdominal fibromatosis, which occurs in the shoulder (9), pelvis (10), chest (11) and neck muscles (12) and other muscle regions (13); intraabdominal fibromatosis, which involves the colon (14), small intestinal mesenteric connective tissue (15, 16), and retroperitoneal space (17); and fibromatosis, which can also occur in the abdominal wall (18) (**Table 1**). Two to six cases are newly diagnosed per million people worldwide every year, and more than 90% of DF cases are sporadic and associated with β-catenin gene (CTNNB1) mutations. A small number of DF patients are diagnosed with germline APC mutations, which present as familial adenomatous polyposis (FAP) (19). The incidence of DF in FAP patients is estimated to be 3–30% (20). However, approximately 8% of patients with sporadic DF have a family history of colon cancer, suggesting a genetic predisposition to both diseases (21).

**Table 1:** Treatment and outcomes of invasive fibromatosis in different locations.

Type	Location	Treatment	Outcome	Reference
Extraabdominal fibromatosis	Shoulder	Surgical wide en bloc resection of the tumor	Uneventful recovery	(9)
	Pelvis	Surgical resection	Uneventful recovery	(10)
	Chest	The modified Grunenwald method and Trap Door method; adjuvant radiation	Without recurrence at 14 months after surgery	(11)
	Neck muscles	Function-preserving surgery followed by radiotherapy	Continuous close follow-up and no local recurrences in the past 7 years	(12)
	Other muscle regions	En bloc excision with instrumented fusion followed by local radiotherapy	Successful treatment	(13)
Intraabdominal fibromatosis	Colon	Conservative therapy and physiotherapy	General improvement in signs and symptoms	(14)
	Jejunal mesentery	Exploratory laparotomy and en bloc resection of mesenteric mass, jejunum (with primary anastomosis), and antimesenteric part of 4th portion of duodenum (Heineke–Mikulicz strictureplasty)	Regular follow-up in the gastroenterology and surgery clinic	(15)
	Ileal mesentery	Complete tumor resection	High5-year recurrence-free survival rate and careful follow-up	(16)
	The retroperitoneal space	Tumor resection, right salpingo-oophorectomy, ureterectomy, and ureterocystostomy; pharmacotherapy with tranilast	The mass disappeared	(17)
Abdominal wall		En bloc resection of the underlying musculature (right obliquus externus abdominis, obliquus internus abdominis, transverses abdominis, and rectus abdominis), right testis, and spermatic cord;abdominal wall defect repaired with a polypropylene mesh	Good health and shows no symptoms or imaging signs of recurrence at 20 months postoperative; physical examination revealed the swelling of the right lower extremity	(18)

Computed tomography (CT) and magnetic resonance imaging (MRI) are the main imaging examination methods for detecting DF (22). With respect to the underlying mechanism of DF, the constitutive activation caused by the mutation of the β-catenin oncogene CTNNB1 (in sporadic cases) (23) or the germline activation of the adenomatous polyposis coli (APC) gene (in FAP patients) (24) will activate the Wnt pathway, thus preventing the degradation of cytosolic β-catenin. Therefore, dysregulation of the Wnt pathway plays a key role in the development of a variety of DFs. Moreover, the Wnt pathway is directly related to the pathogenesis of DF, but crosstalk with the Notch signaling pathway is crucial. Notch receptors are transmembrane proteins that play key roles in cell formation, differentiation, and apoptosis (22). In FAP patients, β-catenin-mediated upregulation of jagged-1/2 (jag-1), a notch-specific ligand, has been shown to activate Notch signaling (25). Therefore, the Wnt pathway and the Notch signaling pathway can interact with each other, leading to the occurrence of DF.

The etiology and pathogenesis of retroperitoneal invasive fibromatosis are not fully understood, but it has been reported that, in addition to the activation of abnormal Wnt signaling at the molecular level, which is mediated by the APC/β-catenin pathway and the activation of the Notch signaling pathway, it is related to trauma (including previous surgery), long-term estrogen use, pregnancy or puerperium (26). Studies have revealed that when DF occurs in the retroperitoneum, it usually has a clinical background of familial polyposis coli/familial adenomatous polyposis (FAP), colon cancer or Gardner syndrome, especially when the patient has a history of abdominal surgery (4). Postoperative development of a fibroma may be mistaken for tumor recurrence, especially when the tumor appears at the previous surgical site. It can be local or regional but never metastasizes. The incidence of this type of invasive fibroma is extremely low, and its etiology is unknown (27). Studies have revealed that fibromatosis tends to exhibit local invasive growth and recurrence. The growth is not constant and may eventually subside, but it will also grow rapidly in some periods. It is generally believed that the invasive growth of these tumors is triggered during surgery, particularly during abdominal surgery (27, 28), but the specific mechanism is not clear.

Owing to the rarity of this disease, the clinical diagnosis of DF is not simple, and the misdiagnosis rate can be as high as 30% to 40% (1). On CT images, fibromas usually appear as dense soft tissue masses with clear boundaries and uniform enhancement, but in some cases, they may appear more aggressive with unclear margins (29). Histological diagnosis usually requires the exclusion of other possible mesenchymal tumors, such as gastrointestinal stromal tumors or low-grade leiomyosarcomas (30), and fibromas are characterized by monoclonal fibroblast proliferation, manifested as small bundles of spindle cells in abundant fibrous stroma with low cell density and no malignant characteristics (2). Different types of soft tissue tumors have different immunohistochemical markers, in addition to differences in imaging and histology. Distinct immunophenotypic markers aid in differentiating these tumor types. For example, aggressive fibromatosis (a fibroblastic/myofibroblastic tumor) is characterized by diffuse cytoplasmic and nuclear β-catenin positivity along with vimentin and Ki-67 expression (31). Notably, other fibroblastic tumors lack nuclear β-catenin expression.

The Ki-67 index, widely used as a proliferation marker in clinical practice, reflects tumor cell proliferative activity through its expression in the G1, S, G2, and M phases (absent in the G0 phase), with higher values indicating greater malignant potential (32, 33). SMA and desmin positivity is typically indicative of leiomyoma (34). ALK positivity typically suggests an inflammatory myofibroblastic tumor (35), whereas liposarcoma (the most common soft tissue sarcoma) frequently harbors MDM2 and CDK4 amplifications in chromosomal region 12q13–15 (36). Coexpression of CD34, Bcl-2, CD99, and STAT-6 supports the diagnosis of solitary fibrous tumor (SFT) (37), whereas peripheral nerve sheath tumors are positive for S-100 and SOX-10 (38). Gastrointestinal stromal tumors (GISTs) typically express CD117 and DoG-1 (39). Carcinosarcoma, a biphasic malignancy containing both epithelial and mesenchymal components, is characterized by concurrent CK and vimentin expression (40).

In this case, MRI examination was declined by the patient. We comprehensively summarize the following immunohistochemical profile of this case: β-catenin (+), vimentin (+), Ki-67 (2%), SMA (-), ALK (-), MDM2 (-), CDK4 (-), CD34 (-), Bcl-2 (-), STAT-6 (-), S-100 (-), SOX-10 (-), CD117 (-), DoG-1 (-), and CK (-). This immunoprofile strongly supports the diagnosis of aggressive fibromatosis.

Every year, there are 2 to 6 newly diagnosed cases per million people worldwide, with over 90% of DF cases being sporadic and related to mutations in the β-catenin gene (CTNNB1). To demonstrate the relationship between tumor occurrence and gene mutations, tumor slices were sent for testing and subjected to WES. These results were interpreted based on the 2015 Gene Sequence Interpretation Guidelines and genome coordinates published by the American Society for Medical Genetics and Genomics (ACMG). We have detected a mutation in the CTNNB1 gene, which is located on chromosome 3 at 3p22.1. The coordinates of the gene mutation are located at chr3:41266124 (hg19), and the nucleotide at position 121 of the gene coding region has changed from A to G (c.A121G). Affects four transcripts, namely NM_001098209, NM_001098210, NM_001904, and NM_001330729. This mutation is a missense mutation in all transcripts, causing the 41st amino acid of the CTNNB1 encoded protein β-catenin to change from threonine to alanine (p.T41A), disrupting the GSK3 β phosphorylation site (a key site in the degradation signaling pathway), preventing the normal degradation of β-catenin, leading to abnormal protein accumulation and sustained activation of the Wnt signaling pathway, promoting cell proliferation, and ultimately leading to cancer. According to the ACMG classification, this mutation is defined as PP5 (pathogenicity, ClinVAR database) and PM2 (extremely low population frequency, consistent with carcinogenic mutation characteristics). Therefore, we believe that this disease can enrich our understanding of this type of tumor.

This type of tumor is difficult to treat, and many factors, such as age, tumor location and size, affect the prognosis. Among the many types of DFs, abdominal wall DFs have the best prognosis, followed by intra-abdominal DFs and limb DFs, which are known to have a greater risk of progression. Retroperitoneal invasive fibromas can be divided into three categories: asymptomatic resectable tumors, symptomatic resectable tumors, and unresectable and recurrent tumors. For asymptomatic patients, a “watch and wait” approach may be the most appropriate treatment, as such an approach can avoid unnecessary morbidity caused by surgery or radiotherapy. Surgical resection may be required to prevent disease progression, suggesting that a personalized, multidisciplinary approach to treatment is needed (22). For symptomatic retroperitoneal fibromas, their sensitivity to chemotherapy and radiotherapy is limited, and surgery is still the only curative treatment; however, studies have revealed that incomplete resection of DFs is associated with postoperative progression (41).

In the third category of unresectable and recurrent tumors, treatment options include antihormone therapy, NSAIDs, tyrosine kinase inhibitors, cytotoxic chemotherapy, radiotherapy, and close observation (42). Regardless of the type, this type of fibroma grows slowly, and patients often recover well. Although the prognosis of invasive fibromas after treatment is good, there is no reliable measure for preventing the development of an invasive fibroma after abdominal surgery.

In this case, the patient had a history of colon cancer and abdominal surgery. The invasive fibroma developed within a very short time after surgery. β-catenin showed characteristic nuclear positivity. Meanwhile, the WES results showed a mutation in the CTNNB1 gene. After laparoscopic resection, the patient recovered well without any symptoms or discomfort after the operation. Moreover, we followed the patient, and 2 years later, abdominal computed tomography (CT) revealed no recurrence of the retroperitoneal tumor or the colon cancer. The patient had returned to his preoperative weight.

## Conclusion

Since invasive fibromas in the retroperitoneum are rare, it is essential to report any cases encountered. In this study, we present follow-up data for a patient who developed an invasive fibroma after colon cancer resection surgery. The CT image of the patient showed fibroma formation, with characteristic nuclear positivity of β-catenin immunohistochemistry. At the same time, WES results confirmed a mutation in the CTNNB1 gene. Retroperitoneal invasive fibromatosis is a medium-sized soft tissue tumor characterized by monoclonal fibroblast proliferation, and its clinical course is variable and unpredictable. However, few clinical trials have focused on such patients, and most studies are case series with relatively small numbers of patients. Therefore, more research on such tumors is needed.

## Data Availability

The original contributions presented in the study are included in the article/supplementary material. Further inquiries can be directed to the corresponding author.

## References

[1] Shen C Wang C Yan J He T Zhou X Ma W . Clinicopathological characteristics, treatment, and survival outcomes of retroperitoneal desmoid-type fibromatosis: A single-institution experience in China. Med (Baltimore). (2019) 98:e18081. doi: 10.1097/MD.0000000000018081, PMID: 31764841 PMC6882633

[2] Aidid F Aichouni N Afilal I Abbou W Jabi R Miry N . Retroperitoneal desmoid tumor in a patient with familial adenomatous polyposis: A case report. Radiol Case Rep. (2022) 17:2910–4. doi: 10.1016/j.radcr.2022.05.013, PMID: 35755096 PMC9218292

[3] Meazza C Bisogno G Gronchi A Fiore M Cecchetto G Alaggio R . Aggressive fibromatosis in children and adolescents: the Italian experience. Cancer. (2010) 116:233–40. doi: 10.1002/cncr.24679, PMID: 19950127

[4] Mishra DP Rout SS . Desmoid tumors: A clear perspective or a persisting enigma? A case report and review of literature. World J Oncol. (2016) 7:21–7. doi: 10.14740/wjon961w, PMID: 28983359 PMC5624685

[5] Peng PD Hyder O Mavros MN Turley R Groeschl R Firoozmand A . Management and recurrence patterns of desmoids tumors: a multi-institutional analysis of 211 patients. Ann Surg Oncol. (2012) 19:4036–42. doi: 10.1245/s10434-012-2634-6, PMID: 22972507 PMC3568525

[6] Keusch CF Bauer J . Mesenteric fibromatosis in Gardner’s syndrome. Mt Sinai J Med. (1989) 56:318–20., PMID: 2677697

[7] El Charif MH Tarhini H Dushfunian D Al Harake H Khasawneh H Abi Saad G . Retroperitoneal desmoid-type fibromatosis: a case report. Ann Med Surg (Lond). (2023) 85:1258–61. doi: 10.1097/MS9.0000000000000491, PMID: 37113969 PMC10129236

[8] Ferenc T Sygut J Kopczyński J Mayer M Latos-Bieleńska A Dziki A . Aggressive fibromatosis (desmoid tumors): definition, occurrence, pathology, diagnostic problems, clinical behavior, genetic background. Pol J Pathol. (2006) 57:5–15., PMID: 16739877

[9] Limaiem F Gharbi MA Boujelbene N Triki R Romdhane KB Bouzidi R . Desmoid-type fibromatosis in an uncommon location: A case report of shoulder involvement misdiagnosed as rhabdomyosarcoma. Int J Surg Case Rep. (2024) 125:110508. doi: 10.1016/j.ijscr.2024.110508, PMID: 39461132 PMC11542472

[10] Begum SA . Surgical management of desmoid tumor of the female pelvis: A case report. Mymensingh Med J. (2016) 25:580–4., PMID: 27612912

[11] Noda D Abe M Takumi Y Anami K Miyawaki M Takeuchi H . Resection and postoperative radiation therapy for desmoid fibromatosis of the chest wall in a young woman. Surg Case Rep. (2021) 7:28. doi: 10.1186/s40792-020-01006-5, PMID: 33471222 PMC7817733

[12] Avinçsal Ö M Shinomiya H Otsuki N Sasaki R Nibu KI . Successful management of aggressive fibromatosis of the neck: A case report. Balkan Med J. (2018) 35:278–81. doi: 10.4274/balkanmedj.2017.0509, PMID: 29843498 PMC5981128

[13] Yogesh Kumar B Vidyadhara S Vadhiraja BM . Pediatric recurrent aggressive spinal fibromatosis with progressive kyphosis and neurological deficits. J Orthop Surg (Hong Kong). (2019) 27:2309499019846618. doi: 10.1177/2309499019846618, PMID: 31079576

[14] Aljahdali NF Alolah AA Alghamdi AA Alharthi FF Aljaziri NJ . Fibromatosis colli: A case report. Cureus. (2023) 15:e47308. doi: 10.7759/cureus.47308, PMID: 38022171 PMC10657154

[15] Huang S Shah JM Quintero E Xiao P Asarian A Reddy M . Distal duodenal stricture secondary to mesenteric fibromatosis (Intra-abdominal desmoid tumor) of the jejunum. Case Rep Gastroenterol. (2024) 18:231–7. doi: 10.1159/000538489, PMID: 38645406 PMC11032182

[16] Kuwabara H Katayanagi S Koganezawa I Nakagawa M Katsumata K Tsuchida A . Sporadic intra-abdominal desmoid tumor with a very unusual onset: two case reports. J Med Case Rep. (2021) 15:457. doi: 10.1186/s13256-021-03058-z, PMID: 34526110 PMC8444561

[17] Ono H Hori K Tashima L Tsuruta T Nakatsuka SI Ito K . A case of retroperitoneal desmoid-type fibromatosis that involved the unilateral ureter after gynaecologic surgery. Int J Surg Case Rep. (2018) 47:30–3. doi: 10.1016/j.ijscr.2018.03.039, PMID: 29705676 PMC5994688

[18] Zhao J Cheng F Yao Z Zheng B Niu Z He W . Surgical management of a giant desmoid fibromatosis of abdominal wall with vessels invasion in a young man: A case report and review of the literature. Front Surg. (2022) 9:851164. doi: 10.3389/fsurg.2022.851164, PMID: 35478728 PMC9037953

[19] Kasper B Ströbel P Hohenberger P . Desmoid tumors: clinical features and treatment options for advanced disease. Oncologist. (2011) 16:682–93. doi: 10.1634/theoncologist.2010-0281, PMID: 21478276 PMC3228186

[20] Bertario L Russo A Sala P Varesco L Giarola M Mondini P . Multiple approach to the exploration of genotype-phenotype correlations in familial adenomatous polyposis. J Clin Oncol. (2003) 21:1698–707. doi: 10.1200/JCO.2003.09.118, PMID: 12721244

[21] Fiore M Rimareix F Mariani L Domont J Collini P Le Péchoux C . Desmoid-type fibromatosis: a front-line conservative approach to select patients for surgical treatment. Ann Surg Oncol. (2009) 16:2587–93. doi: 10.1245/s10434-009-0586-2, PMID: 19568815

[22] Mangla A Agarwal N Schwartz G . Desmoid tumors: current perspective and treatment. Curr Treat Options Oncol. (2024) 25:161–75. doi: 10.1007/s11864-024-01177-5, PMID: 38270798 PMC10873447

[23] Lazar AJ Tuvin D Hajibashi S Habeeb S Bolshakov S Mayordomo-Aranda E . Specific mutations in the beta-catenin gene (CTNNB1) correlate with local recurrence in sporadic desmoid tumors. Am J Pathol. (2008) 173:1518–27. doi: 10.2353/ajpath.2008.080475, PMID: 18832571 PMC2570141

[24] Latchford A Volikos E Johnson V Rogers P Suraweera N Tomlinson I . APC mutations in FAP-associated desmoid tumours are non-random but not ‘just right’. Hum Mol Genet. (2007) 16:78–82. doi: 10.1093/hmg/ddl442, PMID: 17135276

[25] Rodilla V Villanueva A Obrador-Hevia A Robert-Moreno A Fernández-Majada V Grilli A . Jagged1 is the pathological link between Wnt and Notch pathways in colorectal cancer. Proc Natl Acad Sci U.S.A. (2009) 106:6315–20. doi: 10.1073/pnas.0813221106, PMID: 19325125 PMC2669348

[26] Zou RQ Liu F Li FY . Retroperitoneal desmoid-type fibromatosis: Challenges in the preoperative diagnosis and treatment. Asian J Surg. (2023) 46:1270–1. doi: 10.1016/j.asjsur.2022.08.059, PMID: 36064487

[27] Lewis JJ Boland PJ Leung DH Woodruff JM Brennan MF . The enigma of desmoid tumors. Ann Surg. (1999) 229:866–72; discussion 872-3. doi: 10.1097/00000658-199906000-00014, PMID: 10363901 PMC1420834

[28] Campara Z Spasic A Aleksic P Milev B . An aggressive retroperitoneal fibromatosis. Med Arch. (2016) 70:154–7. doi: 10.5455/medarh.2016.70.154-157, PMID: 27147794 PMC4851536

[29] Teo HE Peh WC Shek TW . Case 84: desmoid tumor of the abdominal wall. Radiology. (2005) 236:81–4. doi: 10.1148/radiol.2361031038, PMID: 15987965

[30] Lacka DE Nasierowska-Guttmejer A . Fibromatosis - immunohistochemical evaluation, differential diagnosis from gastrointestinal tumors, and other mesenchymal tumours. Prz Gastroenterol. (2019) 14:79–85. doi: 10.5114/pg.2019.83429, PMID: 30944681 PMC6444105

[31] Leithner A Gapp M Radl R Pascher A Krippl P Leithner K . Immunohistochemical analysis of desmoid tumours. J Clin Pathol. (2005) 58:1152–6. doi: 10.1136/jcp.2005.026278, PMID: 16254103 PMC1770757

[32] Miller I Min M Yang C Tian C Gookin S Carter D . Ki67 is a Graded Rather than a Binary Marker of Proliferation versus Quiescence. Cell Rep. (2018) 24:1105–1112.e5. doi: 10.1016/j.celrep.2018.06.110, PMID: 30067968 PMC6108547

[33] Kreipe H Harbeck N Christgen M . Clinical validity and clinical utility of Ki67 in early breast cancer. Ther Adv Med Oncol. (2022) 14:17588359221122725. doi: 10.1177/17588359221122725, PMID: 36105888 PMC9465566

[34] Campione E Di Prete M Costanza G Saggini A Agostinelli S Terrinoni A . Increased occurrence of cutaneous leiomyomas and dermatofibromas in patients with uterine leiomyomas without fumarate hydratase gene mutations. Dermatopathol (Basel). (2023) 10:231–43. doi: 10.3390/dermatopathology10030032, PMID: 37606484 PMC10443243

[35] Zhao S Liu W Li S Shi T Chen Q Li Q . A case of simultaneously diagnosed lung adenocarcinoma and endobronchial inflammatory myofibroblastic tumor with two distinct types of ALK translocation. Cancer Res Treat. (2021) 53:601–6. doi: 10.4143/crt.2020.952, PMID: 33091968 PMC8053870

[36] Ludwig MP Galbraith MD Eduthan NP Hill AA Clay MR Tellez CM . Proteasome inhibition sensitizes liposarcoma to MDM2 inhibition with nutlin-3 by activating the ATF4/CHOP stress response pathway. Cancer Res. (2023) 83:2543–56. doi: 10.1158/0008-5472.CAN-22-3173, PMID: 37205634 PMC10391328

[37] Fonsêca TC Agostini M Paes JV Roza ALOC van Heerden WFP Romañach MJ . Solitary fibrous tumor of the mandible. Head Neck Pathol. (2024) 18:124. doi: 10.1007/s12105-024-01731-5, PMID: 39589596 PMC11599492

[38] Zhou D Xing X Fan J Zhang Y Liu J Gong Y . PD-1/PD-L1 negative schwannoma mimicking obstructive bronchial Malignancy: A case report. Thorac Cancer. (2020) 11:2335–8. doi: 10.1111/1759-7714.13505, PMID: 32510862 PMC7396376

[39] Hu D Duan Y Chen Y Li B Du Y Shi S . A case report of gastrointestinal stromal tumor of the duodenum. Am J Transl Res. (2022) 14:8279–85., PMID: 36505329 PMC9730101

[40] Wang Z Jiang Z Yang Y Li J Zhang C Liu Z . A case of tonsil sarcomatoid carcinoma. Lin Chuang Er Bi Yan Hou Tou Jing Wai Ke Za Zhi. (2020) 34:183–5. doi: 10.13201/j.issn.1001-1781.2020.02.021, PMID: 32086930 PMC10128419

[41] Nuyttens JJ Rust PF Thomas CR Jr Turrisi AT 3rd . Surgery versus radiation therapy for patients with aggressive fibromatosis or desmoid tumors: A comparative review of 22 articles. Cancer. (2000) 88:1517–23. doi: 10.1002/(SICI)1097-0142(20000401)88:7<1517::AID-CNCR3>3.0.CO;2-9 10738207

[42] Desmoid Tumor Working Group . The management of desmoid tumours: A joint global consensus-based guideline approach for adult and paediatric patients. Eur J Cancer. (2020) 127:96–107. doi: 10.1016/j.ejca.2019.11.013, PMID: 32004793

